# 608 Utilization of Phase-Based Guidelines For Patient Care After Application of Cultured Epithelial Autograft

**DOI:** 10.1093/jbcr/irac012.236

**Published:** 2022-03-23

**Authors:** Kathleen M Ewanowski, Caitlin Bramble, Walter R Anyan, Irma D Fleming, Ronda Hopkins, Stephen E Morris, Callie M Thompson, Giavonni M Lewis

**Affiliations:** University of Utah Burn Center, Salt Lake City, Utah; University of Utah Burn Center, Salt Lake City, Utah; University of Utah Burn Center, Salt Lake City, Utah; University of Utah Burn Center, Salt Lake City, Utah; University of Utah Burn Center, Salt Lake City, Utah; University of Utah, Cottonwood Heights, Utah; University of Utah Health, Salt Lake City, Utah; University of Utah Burn Center, Salt Lake City, Utah

## Abstract

**Introduction:**

Cultured epithelial autografts (CEA) have been clinically utilized since 1981 & can be a lifesaving procedure in patients with extensive full thickness burns. CEA is more susceptible to bacterial contamination & complete graft loss than traditional split-thickness autografts, yet no standard of practice exists for the postoperative care of these grafts to minimize infection & maximize graft take.

Prior to 2019, care of CEA patients at our institution was not standardized & instead varied upon the attending surgeon’s practice. With the input of interdisciplinary team members, CEA patient care was standardized via phase-based guidelines (PBGs), leading to improved team communication & improved patient outcomes.

**Methods:**

PBGs were created via interdisciplinary collaboration among surgeons, APCs, nursing staff, PT/OT, & psychosocial providers. Team members agreed upon 3 facets of patient care: Wound Care/Airing Out, Restrictions/Visitors, & Burn Therapy (Figure 1). As wounds progressed postoperatively, patient phases were advanced, liberalizing them from rigorous infection-prevention techniques to strict unit standards for non-CEA burn patients.

In 2019, the utilization of patient-specific CEA care plans ceased in favor of standardized PBGs. A retrospective chart review was conducted on all patients from 2018-2021 who received CEA & survived their injuries. Some patients underwent a single CEA application while others underwent multiple operations. CEA graft take was assessed on all wounds from each surgery.

**Results:**

CEA was rarely used at our institution. Beginning in 2018, seven patients received CEA & survived their injuries, ranging in age from 4-59 yrs (mean 24) & %TBSA from 38-80 (mean 53) (Table 1). Implementation of PBGs correlated with subjective improvement in team communication & increased mean percentages of CEA graft take from < 35% to >75%.

**Conclusions:**

PBGs have standardized care for our CEA patients, eliminated communication errors among team members, & increased CEA graft take. Further research is needed to determine efficacy in decreasing infection, antibiotic use, hospital stay length, & mortality in these patients.

